# RNA modifications in aging-associated cardiovascular diseases

**DOI:** 10.18632/aging.204311

**Published:** 2022-09-29

**Authors:** Xinyu Yang, Priyanka Gokulnath, H. Immo Lehmann, Zhitao Hou, Sun Yang, Liangzhen You, Guoxia Zhang, Yanwei Xing, Ji Lei, Guoping Li, Shuwen Guo, Hongcai Shang

**Affiliations:** 1 Fangshan Hospital Beijing University of Chinese Medicine, Beijing 102400, China; 2 Key Laboratory of Chinese Internal Medicine of the Ministry of Education, Dongzhimen Hospital Affiliated with Beijing University of Chinese Medicine, Beijing 100700, China; 3 Cardiovascular Research Center, Massachusetts General Hospital and Harvard Medical School, Boston, MA 02114, USA; 4 College of Basic Medical and Sciences, Heilongjiang University of Chinese Medicine, Harbin 150040, Heilongjiang, China; 5 Guang’an Men Hospital, Chinese Academy of Chinese Medical Sciences, Beijing 100053, China; 6 Center for Transplantation Science, Massachusetts General Hospital, Harvard Medical School, Boston, MA 02114, USA

**Keywords:** RNA modifications, aging, aging-related cardiovascular diseases, epitranscriptome

## Abstract

Cardiovascular disease (CVD) is a leading cause of morbidity and mortality worldwide that bears an enormous healthcare burden and aging is a major contributing factor to CVDs. Functional gene expression network during aging is regulated by mRNAs transcriptionally and by non-coding RNAs epi-transcriptionally. RNA modifications alter the stability and function of both mRNAs and non-coding RNAs and are involved in differentiation, development, and diseases. Here we review major chemical RNA modifications on mRNAs and non-coding RNAs, including N6-adenosine methylation, N1-adenosine methylation, 5-methylcytidine, pseudouridylation, 2′ -O-ribose-methylation, and N7-methylguanosine, in the aging process with an emphasis on cardiovascular aging. We also summarize the currently available methods to detect RNA modifications and the bioinformatic tools to study RNA modifications. More importantly, we discussed the specific implication of the RNA modifications on mRNAs and non-coding RNAs in the pathogenesis of aging-associated CVDs, including atherosclerosis, hypertension, coronary heart diseases, congestive heart failure, atrial fibrillation, peripheral artery disease, venous insufficiency, and stroke.

## INTRODUCTION

The incidence of cardiovascular disease (CVD) is increasing rapidly around the world, taking nearly 17.9 million lives every year, and this number will increase to 23.6 million by 2030 [1–3]. This medical burden is expected to increase in developing and developed countries because of the aging population and changing risks factors posed by the environment. During aging, heart and blood vessels gradually exhibit homeostatic imbalances leading to vascular sclerosis and fibrosis, increased left ventricular (LV) wall thickness, decreased tissue fitness, and reduced stress tolerance [4, 5]. These changes contribute to different types of CVDs, including atherosclerosis, hypertension, peripheral artery disease, venous insufficiency, stroke, coronary artery diseases (CAD), atrial fibrillation (AF), congestive heart failure (CHF), and cardiac hypertrophy. Better understanding of the underlying signaling pathways that are involved in these diseases may lead to the development of novel targeted therapies against CVDs. Recent advances in next-generation sequencing, particularly RNA sequencing (RNA-Seq), have enabled the study of new classes of non-coding RNAs (ncRNAs), such as long ncRNA (lncRNA), miRNA, circular RNAs (circRNAs), apart from the traditionally well-known mRNA, rRNA, and tRNAs. Among them, RNA modifications have emerged as one of the key contributors in the pathogenesis of aging-associated CVDs.

More than a hundred RNA modifications have been identified that could alter the chemical and topological properties of the ribonucleotide molecules to perform specific biological functions during their post-transcriptional regulation. Initially, RNA modifications were only studied in tRNA, rRNA, and small nuclear RNAs (snRNA). Eventually, through multiple advanced tools together with subsequent next-generation sequencing [6, 7], even low abundant modifications are now increasingly discovered in most RNAs including mRNA [8], miRNA [9], circRNA [10], lncRNA [11], snRNA, and snoRNA [12]. RNA modifications can directly affect RNA chemistry, including secondary structure, base pairing, and the ability to interact with proteins. Of note, these changes modulate gene expression by regulating RNA processing, localization, translation, and decay [13]. In CVDs, extensive RNA modifications serve as novel mechanisms that underlie the hypertension, CAD, and CHF. In this review, we will summarize the molecular evidence towards the known regulation and interaction of several kinds of RNA modifications in CVDs.

## Different RNA species that undergo post-transcriptional modification

**Figure 1:**
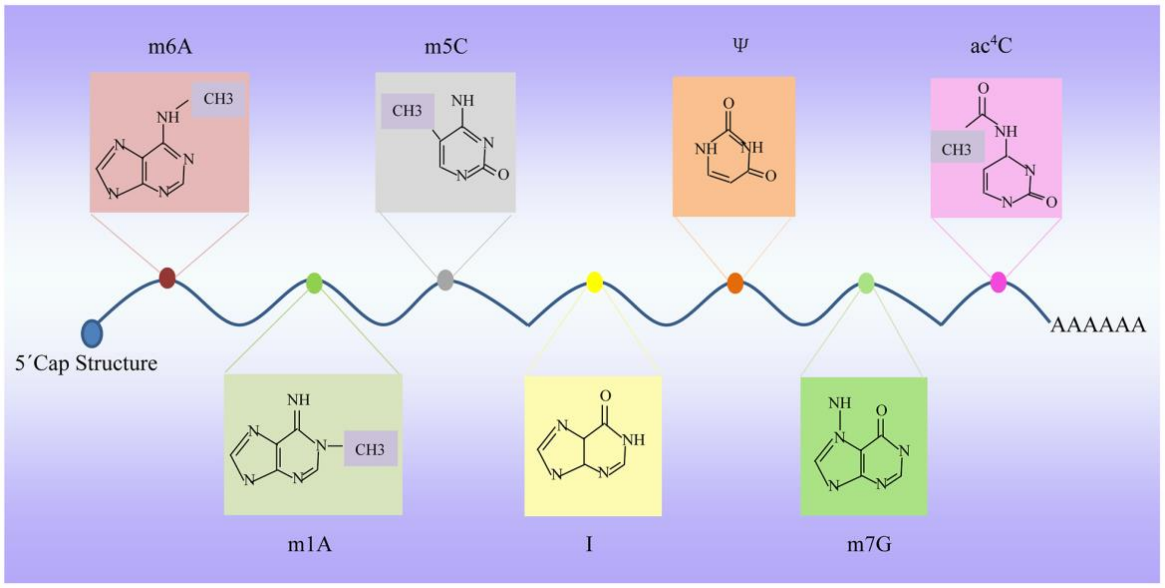
**Dynamic RNA modifications.** Multiple internal modifications within mRNAs, and specific groups of modifications are highlighted, along with modification deposition, removal, and base pairing of modification pairs for downstream recognition. m6A, N6-adenosine methylation; m5C, 5-methylcytidin; ac4C, N4-acetylcytidine; Ψ, pseudouridine; A-to-I, adenosine-to-inosine; m7G, N7-methylguanosine; m1A, N1-adenosine methylation.

RNA is one of the key molecules, that can perform most types of functions in a cell, and dynamic modifications of RNA have been identified in the transcriptome which are largely conserved across species evolutionarily [14]. Therefore, understanding its structure and functional relationship is not only critical for a deeper understanding of basic molecular biology but also has important implications for human health [15]. Most RNA modifications have been primarily identified in mRNAs (Figure 1). In the following sections, we delve deeply into how these different aging-related mRNA modifications impact CVDs. Although there are nearly 170 types of RNA modification, we will only be discussing the topmost frequently observed modifications that influence aging-associated cardiovascular health in this review. In addition to the classical mRNA, the progress of advanced experimental techniques and computational methods have led to the discovery of various ncRNAs species of different lengths and biological roles that undergo RNA modifications as well. We will also briefly discuss those that are critically involved in aging-associated CVDs.

RNAs other than protein coding mRNAs are literally categorized as ncRNAs. For the ease of understanding, ncRNAs are further classified into two categories based on their function. The first class is “infrastructural RNAs” that involve in basic housekeeping functions, such as protein-coding related RNAs including RNAs designated for the protein translation, namely rRNA and tRNA, as well as RNAs responsible for their maturation and even splicing, including snRNAs and snoRNAs [16]. SnRNAs mainly play a role in pre-mRNA processing in the nucleus and snoRNAs have an impressive diversity of functions including rRNA and snRNA modifications [17].

The second class of ncRNAs includes regulatory RNAs that include all other cellular ncRNAs not included as infrastructural RNAs. For instance, short ncRNAs include miRNAs, which can alter gene expression by degrading mRNA molecules after transcription, while the piwi-interacting RNAs (piRNAs) silence retrotransposons in the germ cells [18]. A lesser known, enhancer RNA (eRNA) has been reported to be a ncRNA molecule transcribed from the enhancer regions that is involved in transcription regulation [19]. They are 100-9000 nucleotides long synthesized from regions enriched with H3K4me, RNA Polymerase II and co-activators such as p300. They are believed to be transcription activators and since they have only recently been identified, their mechanism of action is still unclear. Promoter Associated RNA (PAR) are 16-200 nucleotides in length and are usually observed to be expressed around the transcription start site or near the promoter, preferentially near GC rich regions of highly expressed genes. They are usually poorly expressed with short half-lives and are observed to be involved in transcriptional regulation. Another vast and important type of ncRNAs are the lncRNAs. lncRNAs are defined as being over 200 nucleotides in length, some even exhibiting mRNA-like characteristics, such as being 5’-capped, spliced and undergoing polyadenylation. They are transcribed in tissue-specific, disease-specific, and developmental regulatory ways, mediating epigenetic changes by enrolling chromatin remodeling complexes to specific genomic sites along many of their other functions. CircRNAs are usually 4-6 exon long evolutionarily conserved closed continuous RNA loops without any poly A tails, principally acting as miRNA sponges. More recently, they have increasingly been observed to have important physiological roles such as in cancer and CVDs.

## Detection of dynamic RNA modifications

While techniques such as chromatography or mass spectrometry could recognize many extensive modifications, more rare RNA modifications require advanced sequencing techniques to identify the nucleosides that have undergone modifications and differentiate these from unmodified ones [20, 21]. Identification involves several challenges. For example, in the process of reverse transcription (RT), the modifications may interfere with the production of reverse transcribed copies of cDNA. Recent studies have revealed that the N1-methyladenosine (m1A) modifications in the template RNA has an influence on the cDNA synthesis and their application in the test of their respective patterns in the machine learning-based high-throughput sequencing data [22]. In this section, we will introduce some of the techniques that are commonly used to identify most RNA modifications [23] (Table 1).

**Table 1:** Techniques for detecting RNA modifications.

**Method**	**Application**	**Main pathway of action**	**Detection sensitivity**	**Reference**
**Mass spectrometry**	various modifications	the fragment mode	Low (5 μg)	24
**LC-MS**	various modifications	complementary DNA oligonucleotides	High (0.5 μg)	27, 28
**Antibody-based enrichment techniques**	m6A and m7G	Antibodies recognizing m6A or m7G modification	High (1 μg)	31, 32
**Modifications in high throughput sequencing**	Ψ and I	RT arrest or misincorporation	High (10 ng-1 μg)	34
**Bisulfite treatment**	m5C	bisulfite based on RNA	High (5 ng - 1 μg)	37, 38
**MeRIP**	2'-O-Me	the 5ʹ and 3ʹ linker connection protocol	High (1 μg -2 μg)	39
**SCARLET**	m6A and Ψ	RNase H	High (1 μg)	40, 41
**RiboMethSeq**	2'-O-Me	RT under low concentration dNTP	High (1 μg)	43

LC-MS, Liquid chromatography-mass spectrometry; RT, reverse transcription; MeRIP, Methylated RNA Immunoprecipitation; RiboMethSeq, ribose methylation sequencing.

### Mass spectrometry

It is the most direct method for the high-throughput and sequence-specific analysis of the transcriptome stems from an adaptive mean widely used in proteomics [24]. The elution fragments are analyzed by mass spectrometry and the modification can be detected from the fragment mode by comparing the calculated quality with that of the unmodified fragment [25]. This approach has been used to RNAs. Although nucleases (RNase T1 and MC1) permit the preparation of fragment libraries, protocols, tools, and databases common in proteomics are largely lacking in the RNA domain.

### Modifications in liquid chromatography-mass spectrometry (LC-MS)

In this technique, nuclease protection was achieved by forming double-stranded bodies with complementary DNA oligonucleotides, such that the unhybridized RNA was then degraded by the nucleases and the remaining double-stranded bodies were analyzed by the LC-MS for nucleoside analysis [26, 27]. Other methods that had been used to excise certain fragments from larger RNAs including the site-specific cleavages applied to the RNase H and short DNA oligonucleotides or enzymes [28]. The retention behavior utilized in the LC-MS method reflected a biophysical property that also formed the basis of thin layer chromatography (TLC) that is applied to identify and quantify in the RNA modifications.

### Antibody-based enrichment techniques

These are very useful tools known for their very high affinity and the potential to specifically recognize molecular structures [29]. Nucleic acid applications include the analysis of DNA modifications and the generation of specific antibodies against the modified nucleotides in the RNA with a long history [30, 31]. Meanwhile, more antibodies to m6A, m1A, m5C and hm5C are currently available. Because antibody binding provides only the non-covalent complexes with modified RNA, the rigor and enrichment of the washing step is limited. Significant improvement was achieved by an ultraviolet (UV)-induced cross-linking step after the formation of a non-covalent complex between the modified RNA and the antibody [31, 32]. In addition, even single-nucleotide resolution was achieved using this technique by analyzing the unique signal generated due to the covalent cross-linking of a specific antibody to a particular RNA modification that in turn leaves a specific RT signature in the corresponding sequencing spectrum [33].

### Modifications in high throughput sequencing

Another dimension of information can be accessed using high-throughput sequencing data after processing RNA templates with reagents that can react specifically with the modifications to alter their RT characteristics in the terms of RT stasis or misincorporation. For example, the inosine specific cyanoethylation (ICE-SEQ) of acrylonitrile produces a strong RT termination that can distinguish true A to I conversion sites from simple sequencing errors [34]. Currently, the well-known reaction specificity of the ψ residue of N-cyclohexyl-N'-(2-morpholine ethyl) carbodiimide methyl-p-toluene sulfonic acid (CMCT) has been applied in multiple independent pseudouridine (PU) mapping in the transcriptomes of yeast and human. [35, 36].

### Bisulfite treatment

Recent adaptation for the detection of m5C in bisulfite based on RNA. In the process of this technology, the technology was based on the mature detection of 5-methylcytosine (5mC) and 5-hydroxymethylcytosine (5hmC) in the DNA, and the modification of cytosine to m5C or 5hmC can protect these sites from the impacts of bisulfite treatment [37, 38].

### MeRIP

In the MeRIP protocol or ribose methylation sequencing (RiboMethSeq) method, the precise definition of the fragment is located in the 5ʹ and 3ʹ linker connection protocol [39]. The ribose 2ʹ-O-methylation in the yeast rRNA was obtained by two published RiboMethSeq methods and compared with data acquired by direct rRNA performed by the mass spectrometry.

### SCARLET

The latest and most complex development is the SCARLET variation. In the SCARLET method, the target RNA was first cleaved with RNase H at the desired site and the cleavage was guided by the chimeric oligonucleotides containing DNA and 2ʹ -O-Me-RNA [40, 41]. In several applications, it has been applied to verify the high-throughput data for the m6A and ψ residues in the mRNA and lncRNA.

### RiboMethSeq

In some cases, RT primer extension can also be useful as a verification method for high-throughput mapping candidates of the RT arrest signals [42]. One example is the modification after alkaline hydrolysis according to the RiboMethSeq method, which is verified by RT under low concentration dNTP [43]. Invalid cDNA produced at the 2ʹ -o-methylation site can be analyzed directly by the polyacrylamide gel electrophoresis (PAGE) or semi-quantitative PCR or qPCR analysis.

## Bioinformatic tools and resources in epitranscriptomics

Several epitranscriptome-wide landscapes of RNA modifications have been performed using high-throughput sequencing and these have led to the development of extensive databases for such modifications. They are important tools in furthering this field and have been discussed as shown below:

### Databases for RNA modifications

The following are some common RNA modification databases: MODOMICS-RNA modification pathway database [44], RNA modification database (RNAMDB) [45], MeT-DB-mammalian cell transcriptome methylation database [46], and a database devoted to RNA modification in the normal and disease, such as the RNA Modification Base (RMBase) [47].

### MODOMICS

The MODOMICS database is a reference database for RNA modification because it provides the most complete information on the chemical structure of the modified ribosides, reaction abstracts, functional characteristic enzymes involved in the modification, and the biosynthetic pathways of RNA modifications [44].

### RNAMDB

As a reference for updating the RNA modification results, the RNAMDB portal provides many useful tools for the mass spectrometry identification of natural or modified RNAs. Beginning with RNA sequences, molecular weight, electrospray series, CID fragment, internal fragment, base loss, and fragment digestion can be calculated [45].

### MeT-DB

The MeT-DB is a synthetical database focused on the m6A mammalian methyl transcriptome. It includes approximately 300,000 m6A methylation sites, which have been detected in the samples from humans, mice, and cells under various experimental conditions [46]. Data were analyzed by the methylated RNA immunoprecipitation sequencing (MERIP-SEQ) and detected by the exomePeak and MACS2 algorithms [48, 49].

### RMBase

The RMBase is also a comprehensive database. It integrates epigenome sequencing data to explore post-transcriptional modification of RNAs and their relationships with the miRNAs, the disease-associated the RNA-binding proteins (RBPs) and single nucleotide polymorphisms (SNPs) [50]. Meanwhile, RMBase has provided various interfaces and graphical visualizations to facilitate the analysis of a large number of modification sites.

### Bioinformatic tools to predict RNA modifications

The computer methods development based on the support vector machines, which can accurately predict post-transcriptional modification sites from sequence information, is very helpful for the scientific community to further understand epigenetic modification [51, 52]. As a good complement to experimental innovations, many computational methods have been put forward in recent years to forecast the RNA modification sites. Some of the currently available online calculation tools for predicting RNA modification sites, are HAMR, PAI, iRNA-AI, RAMPred, iRNA-3typeA, iRNA-PseColl, iRNAm5C-PseDNC, iRNA-Methyl, m6Apred, MethyRNA, SRAMP, RAM-ESVM, PPUS, iRNA-PseU, tRNAMOD [52–67].

## Epigenetically regulated RNA modification in aging-related cardiovascular diseases

Aging is viewed as a multifactorial process that derives from the interaction of genetic and environmental factors. It can be divided into pathological aging and physiological aging. Physiological aging is a complex biological process characterized by the decline of tissue and organ function, structural degradation, and decline in adaptability and resistance, all of which led to an increase in the morbidity and mortality of many chronic diseases [68, 69]. The changes of cardiovascular aging principally include LV hypertrophy, increased cardiac fibrosis, AF and arteriosclerosis, diastolic dysfunction, and maximum exercise capacity and decreased arterial compliance [70, 71]. Cardiovascular aging makes the heart more sensitive to stressors, such as, smoking, hypertension, hypercholesterolemia, diabetes and other cardiovascular risk factors [70]. This is more prevalent and has to be approached differently as opposed to cardiovascular disorders driven by congenital or other genetic causes. While congenital disorders do contribute to CVDs, it is the aging-associated CVDs that attribute to most mortality as well as healthcare burden. An increase in the age-related markers of inflammation may be responsible for the reduced ability of older organisms to deal with various stressors. Inflammation may also produce reactive oxygen species (ROS) that leads to oxidative damage and induce increased cytokine release, thereby exacerbating the vicious cycle of simultaneous activation of tissue damage and repair mechanisms, resulting in a chronic pro-inflammatory state [72, 73]. Over decades, this damage has accumulated slowly and asymptomatically, leading to the development of age-related diseases, such as cardiovascular disease [74]. A better understanding of cardiovascular aging will contribute to delayed progression and thus decrease the adverse consequences of cardiovascular disease. In fact, it is necessary to enumerate the different aging associated biological changes because CVDs account for nearly 40% of all aging related disorders and is the largest contributor among aging related diseases [75].

Cardiovascular aging can be roughly categorized into four diverse groups of disorders based on their pathogenesis [76, 77]:

- Changes in cardiac structure due to aging – Aging leads to molecular and cellular changes, ultimately leading to compromised functional changes such as decrease in adaptive capacity of cardiac cells and cardiomyocyte loss [3].
- Aging promoted epigenetic cardiovascular changes – Epigenetic changes such as DNA methylation changes, post-translational histone modifications, and chromatin remodeling changes have been observed to be important factors in development of CVD [77].
- Autonomous Nervous System (ANS) – The effect of aging can irreversibly affect the ANS, which is known to have an important role in cardiovascular homeostasis and can in turn have an adverse impact on the cardiovascular system [78].
- Development of certain CVDs – Aging itself is a known risk factor for several CVDs that develop in an aging body such as cardiac fibrosis, hypertrophy, diabetes, hypertension, atherosclerosis, ischemic injury, MI, stroke, etc. [79, 80].

**Figure 2:**
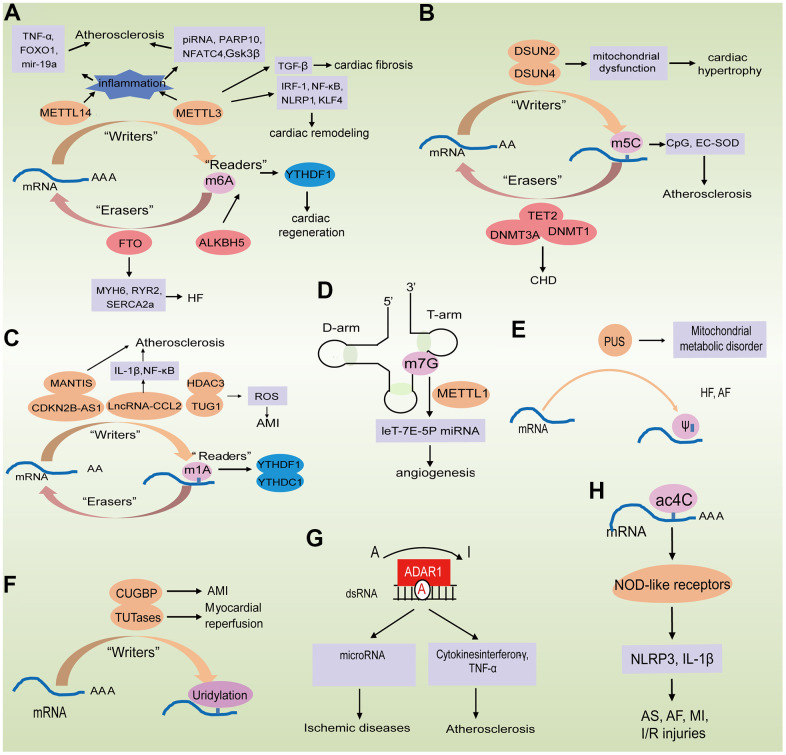
**Epigenetically regulated RNA modification in aging-related cardiovascular diseases.** (**A**–**C**) The biological functions of m6A, m5C and m1A are mediated by writer, eraser, and reader proteins; (**D**) The role of m7G in mRNA biogenesis in processing and repression of migration; (**E**, **F**) The biological functions of PU and uridylation are mediated by writer proteins; (**G**) A-to-I RNA editing of ADAR modified the mRNA; (**H**) Potential role of Ac4C in improving mRNA stability.

The study of RNA provides new molecular insights into cardiovascular aging and can serve as a short-term repository for the genetic information in the form of mRNA, or as a long-term repository in form of viral genomes [81]. siRNA, piRNA and miRNA can directly adjust the expression levels of other RNA entities, and their corresponding protein products [18]; tRNA and rRNA can be used as physical scaffolding for binding proteins to amino acids [16, 82], whereas ribozymes such as rRNA and spliceosomal RNA can directly catalyze basic biochemical reactions [17, 82]. RNA entities perform extremely diverse biological roles, which are unmatched by other important molecular categories [83]. Among the ncRNAs, miRNAs and lncRNAs have been the most extensively studied under different backgrounds, including physiological processes and disease conditions associated with aging [84], and adjusting gene expression at the transcriptional and post-transcriptional levels through a variety of mechanisms [85–87]. Therefore, elucidating their role in chronic and multifactorial diseases, such as those affecting the cardiovascular system, is challenging. Epigenetic modifications are divided into three interrelated categories: DNA methylation, RNA-based mechanisms including microRNA and non-coding RNA, and post-translational histone modifications [88, 89]. A new field of epigenetics includes RNA-based mechanisms, including gene regulation via lncRNAs. There is increasing evidence that changes in RNA modification are related to the pathogenesis of CVD [90]. In the following sections we enumerate the different RNA modifications observed in coding and non-coding RNA in the context of aging-driven cardiovascular disorders (Figure 2 and Table 2).

**Table 2:** Roles of RNA modifications in aging-associated cardiovascular diseases.

**Symbol**	**Gene symbol**	**Expression**	**Main target**	**Functions**	**Model**	**Disease**	**Total report #**	**References**
**m6A in mRNA**							90	
	METTL3	decreased	TGF-β1	cardiac fibroblasts	mice	MI		95
	METTL14	decreased	TNF-α, FOXO1	inflammatory response	mice	Atherosclerosis		99
	ALKBH5	decreased	YTHDF1	cardiomyocyte proliferation	mice	Cardiac regeneration		104
	METTL3	increased	m6A	mRNAs	human, mice	Heart failure		96
	METTL3	increased	NF-κB, NLRP1, KLF4	inflammation	endothelial cells	Atherosclerosis		111
	METTL3, METTL4	increased	YTHDF2	ameliorate cardiac function	human, mice	Heart failure		112
	ALKBH5	increased	FTO	cardiac systolic function	mouse	MI		103
**m6A in ncRNA**							35	
	METTL3	increased	IRF-1	macrophage pyroptosis	human	ACS and Atherosclerosis		117
	METTL14	decreased	mir-19a	METTL14/M6A/miR-19a signaling pathway	cells	Atherosclerosis		121
	METTL3	increased	PARP10, NFATC4, GSK3β	CHAPIR–METTL3–PARP10–NFATC4 signaling axis	mice	Cardiac hypertrophy, Cardiac remodeling		126
**m5C in mRNA**							7	
	CpG clusters	decreased	EC-SOD	cellular proliferation and alterations in gene expression	rabbit	Atherosclerosis		133
	TET2	increased	DNMT1, DNMT3A, TET genes	gene expression	human	CHD		136
**m5C in ncRNA**							5	
		bind	YTH domain family proteins	No report	No report	No report		141
**m7G in mRNA**							5	
	METTL1	mediated	CDS and 3'UTR regions	multiple signaling pathways	cells	Vascular diseases		155
**m7G in ncRNA**							2	
	METTL1	mediated	let-7e-5p miRNA	miRNA	cells	No report		157
**PU**		increased	LVMI and RWT	mitochondrial-derived metabolites	human	Heart failure	23	163
		mediated	metabolite levels	metabolize	human	AF		166
**Uridylation**		mediated	CUGBP and CELF	RNA metabolism	mouse	AMI	18	171, 173
**A-to-I in mRNA**							11	
	FLNA	mediated	arterial remodeling, left ventricular wall thickening	vascular contraction and diastolic	human, mice	Hypertension		178
	ADAR1	increased	cytokines interferon-γ and TNF-α	inflammation	cells	Atherosclerosis		179
**A-to-I in ncRNA**							7	
	NEAT1	increased	TNF-α, CXCL8,	inflammation	human	Atherosclerosis		182
			CCL2, VCAM1 and ICAM1					
	ADAR1, ADAR2	increased	miR-376a-3p, miR-376c-3p, miR-381-3p, and miR-411-5p	microRNA	human, mice	Vascular disease		186
**U34**		mediated	mt-tRNA modification enzymes	mitochondrial dysfunctions	cells	Cardiac hypertrophy, mitochondrial diseases	1	194

MI, myocardial infarction; ACS, acute coronary syndrome; AMI, acute myocardial infarction; AF, atrial fibrillation; CHD, coronary heart disease; A-to-I, adenosine-to-inosine; m1A, N1-methyladenosine; m6A, N6-methyladenosine; m5C, 5-methylcytosine; PU, pseudouridine; m7G, 7-methylguanosine; METTL1, methyltransferase-like 1; METTL3, methyltransferase-like 3; METTL14, methyltransferase-like 14; ALKBH5, alkB homolog 5; TNF-α, tumor necrosis factor-α; VCAM1, vascular cell adhesion molecule-1; ICAM1, intercellular cell adhesion molecule-1; GTPBP3, GTP-binding protein isoform V; MTO1, mitochondrial translation optimization 1; CUGBP, CUG binding protein; CELF, CUGBP Elav-Like Family Member; CUGBP Elav-Like Family Member; LVMI, left ventricular mass index; DNMT2, DNA methyltransferase 2; DNMT1, DNA methyltransferase 1; DNMT3A, DNA methyltransferase 3A; TET genes, ten-eleven translocation genes; PARP10, NFATC4, Nuclear Factor Of Activated T-Cells; GSK3β, glycogen synthase kinase-3β; IRF-1, interferon regulatory factor-1; FTO, fat mass and obesity-related protein; YTHDF2, YTH N6-methyladenosine RNA-binding protein 2; NF-κB, nuclear factor kappa-B; KLF4, Krüppel-like factor4; YTHDF1, YTH N6-methyladenosine RNA-binding protein 1; TGF-β1, transforming growth factor-β1; EC-SOD, extracellular superoxide dismutase; ADAR, adenosine deaminases act on RNA.

### N^6^-Methyladenosine modification

### m6A in mRNAs

To date, more than 140 RNA chemical modifications have been reported. Among them, the methylation of mRNA at position N-6 of adenosine residue (m6A) has been the most extensively studied [91]. In many biological processes, this modification makes the mRNA the central hub for intracellular information, acting as an information carrier, modifier, and attenuator. The m6A RNA modification is a reversible and dynamic process that is critical to physiology and pathophysiology [92]. This modification is activated by the methyltransferase complex (“writers”), consisting of methyltransferase-like 3 (METTL3), methyltransferase-like 14 (METTL14), and Wilms tumor-1 associated protein (WTAP). It is removed by an “eraser” consisting of fat mass and obesity-related protein (FTO) and α-ketoglutarate-dependent dioxygenase alkB homolog 5 (ALKBH5). The m6A markers can be recognized by m6A-binding proteins (“readers”), including IGF2BP1-3, YTHDF1-3, and YTHDC1-2.

METTL3 (“writer”) has been reported to be involved in many biological processes associated with disease progression [93, 94]. In fact, it was observed to increase with fibrosis as seen in cardiac fibroblasts, both *in vitro* and *in vivo* fibrosis models with TGF-β1 activation and in MI mouse models respectively [95]. METTL3-mediated m6A modification was critical for the development of cardiac fibrosis and provided molecular targets for the control of fibrosis and related cardiac diseases [96, 97]. Increased m6A methylation has been found in the human cardiomyopathy [98]. The m6A-writing enzyme METTL3 knockdown and overexpression influenced cell remodeling *in vitro* and *in vivo*. These data suggested that the mRNA methylation was highly dynamic in the stressed cardiomyocytes, and that these changes in the mRNA methylation groups could modulate translation efficiency by affecting transcriptional stability [96]. The depletion of METTL3 abrogated the hypertrophy of cardiomyocytes, while increasing METTL3 level led to compensated cardiac hypertrophy [96, 97]. In summary, the METTL3 knockout mice showed morphological and functional of HF induced by aging, suggesting that RNA methylation is necessary for maintaining cardiac homeostasis.

Another study investigated the role of m6A modification in the endothelial cell inflammation and its effect on the atherosclerosis development [99]. Changes in expression of m6A modification-related proteins were assessed using a stable model of tumor necrosis factor –α (TNF-α) –induced endothelial cell inflammation. Methylated RIP sequencing was used to identify m6A modified mRNA, and it was confirmed that METTL14 plays a major role in the TNF-α –induced endothelial inflammation by significantly increasing m6A modification of FOXO1, an important transcription factor for inflammation. Also, the METTL14 knockdown dramatically reduced TNF-α -induced FOXO1 expression [100, 101]. In addition, METTL14 has been indicated to interaction with FOXO1 and directly act on the promoter regions of VCAM-1 and ICAM-1 to accelerate their transcription, thereby mediating the endothelial inflammation [102, 103]. In conclusion, METTL14 promoted the FOXO1 expression by reinforcing the m6A modification and inducing the endothelial inflammatory response and atherosclerosis plaque formation. In fact, the METTL14 could act as a potential therapeutic target for atherosclerosis.

In one study, the role of m6A modification in cardiac regeneration during injury was investigated, and the downregulation of the m6A demethylase ALKBH5 was identified [104]. Mechanically, the ALKBH5-mediated m6A demethylation enhanced the YTH N6-methyladenosine RNA-binding protein 1 (YTHDF1) mRNA stability, thus augmenting its expression and facilitating the YES-related protein (YAP) translation [105]. Similar effects of ALKBH5 and YTHDF1 expression in human induced pluripotent stem cell derived cardiomyocytes were observed [106]. These results emphasized the important effect of ALKBH5-m6A-YTHDF1-YAP axis in regulating CM re-entry into the cell cycle. Another study investigated m6A RNA methylation using the next generation sequencing and discovered that about one quarter of the transcripts from the mouse and human heart existed m6A RNA methylation [107]. These changes in the m6A RNA methylation were observed while looking at the gene expression during the progression of HF in mouse and human [108]. While methylated m6A RNA was primarily connected with regulatory and metabolic pathways, the change in the RNA expression level mainly represented the changes in structural plasticity [109]. Taken together, m6A landscape seems to be altered in cardiac hypertrophy and HF. The disturbance of blood flow caused by the arterial curvature and associated oscillatory stress (OS) at the branch point can activate endothelial activation and is considered one of the risk factors for atherosclerosis. The impact of m6A RNA methylation in the endothelial cell (EC) mechanical transduction has been investigated because of its important role in the regulation of the epigenome [110]. METTL3 was identified as the center of response to hemodynamic forces and atherogenic stimuli in EC. OS could result in METTL3 upregulation causing m6A RNA hypermethylation, enhancing the NF-κB p65 Ser536 phosphorylation, and thereby increasing monocyte adhesion [96]. While METTL3 knockout eliminated OS-induced m6A RNA hypermethylation and other manifestations, its overexpression resulted in changes similar to OS effect. The METTL3-mediated RNA hypermethylation up-regulated NLRP1 transcripts and down-regulated KLF4 transcripts via YTHDF1 and YTHDF2 m6A reader proteins, respectively. *In vivo* atherosclerotic models, partial carotid artery ligation resulted in the plaque formation and up-regulation of METTL3 and NLRP1, and down-regulation of KLF4 [111]. Overall, METTL3 had been shown to play a central role in atherosclerosis caused by OS and blood flow disturbances.

Overall, m6A levels have been reported to be elevated in MI, ischemia-reperfusion injury, and HF with reduced ejection fraction (HFrEF), while reduced m6A may increase autophagy flux and ameliorate cardiac function [103, 107]. In another study, the changes in the heart failure with preserved ejection fraction (HFpEF) patients and mouse models m6A RNA methylation were investigated using the meRIP-seq and the results showed that m6A writers METTL3, METTL4 and KIAA1429; m6A eraser FTO; as well as the reader YTHDF2 were upregulated in HFpEF patients [112] while FTO expression was increased in HFpEF mice [96, 97]. Collectively, given such evidence, m6A methylation might be an important target for therapeutic against CVDs.

Demethylases (“erasers”) include the ALKBH5; FTO which catalyze the demethylation of m6A RNA. One study showed that FTO played a pivotal role in the cardiac systolic function in the process of homeostasis, remodeling, and regeneration [103]. The functional roles of m6A and FTO in the cardiovascular disease were investigated using the human samples, pig and mouse models and cardiomyocyte cultures. FTO expression was reduced in mammalian HF and hypoxic cardiomyocytes, thereby increasing the m6A in RNA and reducing the cardiomyocyte systolic function [113]. Increased FTO expression improved the heart of failing mice through its demethylation activity, which selectively demethylated the cardiac contractile transcripts, thereby preventing their degradation and improving their protein expression [114]. Also, FTO overexpression in MI mouse models was demonstrated to reduce fibrosis and reinforce angiogenesis [115]. Overall, this study demonstrates the FTO-dependent m6A methylation groups functional importance in cardiac contraction during HF, and thus provides novel mechanistic insights into the FTO therapeutic effects.

### m6A in ncRNAs

Pyroptosis is a new type of inflammatory programming cell death, which was recently discovered to be associated with atherosclerosis [116]. A study discovered that interferon regulatory factor-1 (IRF-1) can effectually promote macrophage pyrolysis in the patients with acute coronary syndrome (ACS) [117]. And then there were studies that showed that circRNAs play a role in the atherosclerosis, but their exact mechanism in macrophage pyroptosis remains elusive. The RNA expression of hsa_circ_0002984, hsa_circ_0029589 and hsa_circ_0010283 in the human PCBM-derived macrophages from the patients with CAD was examined. These results showed that the hsa_circ_0029589 relative RNA expression level was reduced in the macrophages, while the N6-methyladenosine (m6A) level and the m6A methyltransferase METTL3 expression was significantly increased in the macrophages of ACS patients. In addition, the overexpression of IRF-1 not only inhibited the hsa_circ_0029589 expression, but induced the m6A and METTL3 expression in macrophages, weakening macrophage pyroptosis [118]. In conclusion, IRF-1 inhibited circ_0029589 by facilitating the m6A modification, consequently promoting the pyroapoptosis and inflammation of macrophages in the ACS and atherosclerosis [119].

In addition, processing of mature mi-RNA has been reported to occur in a m6A dependent manner, thus contributing to the development of pathogenesis [120]. The aim of this study was to demonstrate m6A regulation in atherosclerosis [121]. Methylation expression was assessed using qRT-PCR and indicated that m6A modification levels and METTL14 methyltransferases were significantly overexpressed in atherosclerotic vascular endothelial cells (ASVEC). While METTL14 suppressed proliferation and invasion, its low expression inhibited binding of methylated RNA and RNA splicing related protein DGCR8 in ASVEC. In addition, METTL14 increased m6A modification of pri-mir-19a and accelerated the mature mir-19a processing, thereby promoting the ASVEC proliferation and invasion [122, 123]. Therefore, the METTL14/M6A/mir-19a signaling pathway could be a new therapeutic target for atherosclerosis.

PiRNAs expression changes under various stress conditions, such as cardiac hypertrophy and MI [124, 125]. Gao et al.'s study determined that piRNA promoted cardiac hypertrophy and cardiac remodeling through the METTL3-mediated m6A methylation that targeted the PARP10 mRNA transcripts [126]. The cardiac-hypertrophy-associated piRNA (CHAPIR) deletion dramatically alleviated cardiac hypertrophy and recovered cardiac function [127], while administration of CHAPIR mimics increased pathological hypertrophy of stress overload in mice. In terms of mechanism, the CHAPIR-PIWIL4 compound directly interacts with METTL3 and up-regulates PARP10 expression, thereby blocking m6A methylation [128]. Increased CHAPIR dependence of PARP10 accelerates the mono-ADP-ribosylation of GSK3β, and inhibits kinase activity, and accumulates nuclear NFATC4, thus leading to pathological hypertrophy progression [129]. Therefore, piRNA was involved in regulating of cardiac hypertrophy, and the CHAPIR–METTL3–PARP10–NFATC4 signaling axis could serve as a therapeutic target for the cardiac hypertrophy and cardiac remodeling treatment. Apart from the mentioned above examples, there are several miRNAs and other ncRNAs that undergo m6A modifications to regulate aging.

### 5-Methylcytosine modification

RNA can be methylated at the 5^th^ site of the cytidine residue, which is known as RNA 5-methylcytosine (m5C) discovered in rRNA, tRNA, mRNA, ncRNA, and eRNA [130].

### m5C in mRNAs

Several studies have used diverse techniques to map m5C across the entire transcriptome, identifying m5C abundance in the 8,000 RNAs with only handful mRNAs. However, these differences can be declared by the lack of high specificity m5C antibodies [131]. The m5C methylation on mRNA was shown to be associated with a specific sequence similar to the m5C motif discovered in these tRNAs. In function, the m5C methylation of mRNA was observed to be of great importance for the nuclear output of mature mRNA, mediated by the m5C reader protein and ALYREF [132].

One of the first observations of this modification has been seen in rabbit extracellular superoxide dismutase (EC-SOD) gene which had 5 CpG clusters and 6 C elements [133]. One of the CpG clusters was situated on coded sequence, and a significant reduction in the number of methylated CpG dinucleotides of EC-SOD gene was detected in the atherosclerotic aorta. Further analysis of the genome-wide methylation using highly pressure liquid chromatography uncovered a trend of decreased 5-mC content in the atherosclerotic aortas. This indicates that, the EC-SOD hypomethylation may impact on the structures and functions of EC-SOD and other genes that may be involved in the atherosclerosis occurrence and development [134, 135]. Other studies have explored the relationship between peripheral blood 5-mC levels and the degree of coronary atherosclerosis in the elderly patients with CHD [136]. The 5-mC levels in the peripheral blood mononuclear cells (PBMC) was analyzed in CHD patients and control subjects by dot blot analysis and ELISA. The results suggested that the 5-mC expression level was higher. The expression of TET2 in CHD patients was significantly upregulated, and only the DNMT3A, DNMT1, and all other TET genes expression were discovered increase, and the spearman correlation analysis showed that the 5-mC levels were positively associated with Gensini scores [137–139]. Collectively, 5-mC is a risk factor for CHD, but there is a lack of specific research on the mechanism of RNA modification enzymes.

### m5C in ncRNAs

Several m5C methylation of ncRNA in cardiovascular diseases have been reported. While the m5C methylation on vault RNA influences its processing to derive small RNAs, the m5C in the eRNA preserved them from degradation [140]. The whole-exome sequencing showed that mutations in Nsun2 caused congenital diseases, which in turn affect the heart mainly by causing cardiomyopathy and atrial septal defect. In rRNA, the cardiac-specific KO in NOP2/Sun RNA methyltransferase 4 (Nsun4) mice were associated with mitochondrial dysfunction and cardiomyopathy [141]. In addition, it was well known that tRNA cytosine 5-methyltransferase (Dnmt2) and NOP2/Sun domain family member 2 (Nsun2) are modification enzymes of tRNA m5C that reduced the cleavage of endonucleic acid tRNA [142]. These results showed that the Dnmt2-deficient (Dnmt2^-/-^) mice exhibited myocardial hypertrophy and increased myocardial fibrosis *in vivo* [143]. Meanwhile, the m5C tRNA modification by Nsun2 protected tRNA from the angiogenin-mediated cleavage [144].

### N^1^-Methyladenosine modifications

N1-Methyladenosine (m1A) is another one of the most known RNA modifications in which there is a methyl group added on the N1 position of the adenosine base.

### m1A in mRNAs

The m1A is a significant post-transcriptional modification in the mRNA and can regulate protein translation based on the modification. The various m1A protein readers in RNA that includes several YTH domain family proteins, were identified using quantitative proteomic methods [145]. It was found that YTHDF1and YTHDC3 bound directly to m1A in RNA while YTHDC2 did not. Trp432 in the YTHDF2 was a conserved residue in the of the YTH domain hydrophobic pocket, which was required for combining with m6A, was necessary for the m1A recognition [146]. A previous report that examined the co-localization of proteins containing the YTH domain and the m1A site in the transcriptome, revealed that proteins containing the YTH domain could bind to m1A. Overall, it was found that proteins containing YTH domain could act as m1A readers in RNA, and further research is required to provide newer insights into the function of m1A in RNA biology [147].

### m1A in ncRNAs

The m1A modification is more commonly observed in ncRNAs namely, tRNA, rRNA, and mitochondrial transcripts [147–149]. Just as in m6A methylation, m1A is also reversibly controlled by several transferase complexes and demethylase. While the m1A modification of lncRNA governs its processing, the m1A modification of tRNAs usually stabilizes it [149, 150]. Unlike the m6A levels, there were no significant differences in the m1A levels of urine samples between CHD patients and normal subjects observed in this study [151]. Furthermore, there are no studies that currently associate m1A methylation to CVDs. However, given that among the 22 m1A sites obtained from global sequencing of m1A modification, 10 were observed in 13 mitochondrial transcripts indicating a strong role of m1A in modulating normal mitochondrial function [152].

### Internal 7-methylguanosine

M7G RNA methylation is the seventh N-methylation modification of RNA guanine under the methyltransferase action, mainly including mRNA 5' Cap structure, primary miRNAs (pri-miRNAs), mRNAs, tRNAs and rRNAs. The m7G methylation can regulate mRNA transcription, tRNA stability, miRNA biosynthesis and biological function, and 18S rRNA processing and maturation170-172.

### m7G in mRNAs

In addition to being present at the mRNA cap site, m7G modification is observed in the internal mRNA region [153]. The single-nucleotide resolution cross-linking and immunoprecipitation sequencing (m7G MicLIp-SEQ) was established to test the internal mRNA modification of m7G, and m7G was found to be enriched in the 5'UTR region and AG-rich environment [31]. In addition, m7G modification was dynamically modulated under H2O2 as well as heat shock treatment and had the function of improving mRNA translation efficiency. Results revealed dynamic characteristics of the methylation group of the internal mRNA m7G and highlighted the role of m7G as a novel epigenetic transcriptome marker that regulated translation. Other studies have proven the effect of METTL1-mediated m7G methylation in human induced pluripotent stem cell vascular development. In addition, the METTL1 KO can regulate expression of multiple angiogenesis related genes and mesodermal differentiation and angiogenesis through a variety of signaling pathways [154]. More recently, Zhou et al. have shown that the m7G methyltransferase METTL1 shows promising potential in peripheral arterial disease therapy as this modification was able to increase angiogenesis in post-ischemic injury by increasing the VEGF mRNA translation [155]. Therefore, potential therapies for vascular diseases could target METTL1-mediated m7G modification.

### m7G in ncRNAs

Pandolfini et al. used the Borohydride Reduction sequencing (BoRed-Seq) and RIP to detect m7G in the miRNAs subset that inhibited cells migration. Their study suggested that the METTL1 methyltransferase mediated m7G methylation and regulated cell migration catalytic activity [156]. The m7G was mapped to the single guanosine in the leT-7E-5P miRNA as evidenced by refined mass spectrometry. Another study suggested that METTL1-mediated methylation enhanced processing let-7 miRNA by disrupting the suppression secondary structure in pri-miRNA [154, 156, 157]. Taken together, the METTL1-dependent N7 methylation is a novel RNA modification pathway that modulates miRNA structure and cell migration. For the first time, an extensive transcriptome wide analysis of lncRNAs was performed in hypoxic pulmonary hypertension (HPH) where the m7G lncRNAs were significantly upregulated in HPH compared to non-m7G modified lncRNAs. They also identified two previous unknown lncRNAs with m7G modification that were upregulated, and their roles have been extensively studied to understand their significance in HPH [158]. However, currently there are no other relevant research data regarding this modification or its mechanism in cardiovascular diseases.

### RNA cap methylations

RNA cap methylation or the addition of 7-methylguanosine cap to the first nucleotide of 5’ end is essential for mRNA translation. This addition has also been observed in ncRNAs. This addition of a 5’ cap is also suggested to protect tRNAs from exonuclease activity and makes them less susceptible to degradation. Most diverse modifications are found in tRNA, where cytoplasmic and mitochondrial tRNA carry more than 100 different modifications. Moreover, human tRNA can contain 11 to 13 different modifications, present in different steps of its maturation process, that may directly influence translation. Many of these modifications contribute to stabilizing the tRNA structure. Mutations in human mitochondrial genome [mitochondrial DNA (mtDNA)] lead to multiple clinical diseases; the main unifying feature is altered energy homeostasis due to reduced ATP production.

### Pseudouridine

ψ, also called PU, is the most extensive distributed RNA modification, found among tRNA, rRNA, snRNA, and mRNA [159, 160]. The catalyzed by series of PU synthases (PUS) enzymes that accelerate phosphate skeleton rigidity and increase base stacking, and the unique uridine residues are produced in all kinds of RNAs, mostly in tRNA and rRNA [161]. This process occurs either in an RNA dependent or RNA-independent mechanism. A large number of metabolomic studies have discovered that the PU in plasma and urine is associated with CVDs. Alexander et al. discovered that PU was dramatically correlated with HF. As a result, compared with healthy controls, the plasma PU concentrations were higher in the HFrEF group [162]. Similarly, the elevated plasma PU levels were associated with increased LV mass index (LVMI), and plasma PU combined with natriuretic peptide levels predicted the biomarkers for HFrEF [163]. Thus, PU by itself could be used as a potential biomarker of CVDs.

### ψ in mRNAs

This modification has been reported to be involved in mRNA stability as see in yeast and certain parasites [164]. In fact, RNAs with pseudouridylation of certain mRNAs were observed to increase their abundance. In some cases, pseudouridylation was able to facilitate translation by converting the stop codon to sense codon by modifying certain amino acids. It remains to be seen if this modification also works by modifying bases in the 3’ UTR. PUS genes which are responsible for this modification when undergoing mutations have been reported to lead to mitochondrial myopathies [165].

### ψ in ncRNAs

Pseuduridylation is observed in several types of ncRNA including snoRNAs, telomerase RNA (with a known impact in aging), other low abundant RNAs like rRNAs, snoRNA, intronic RNAs, lncRNAs, RNAse MRP, Steroid Receptor activator, 7SK RNA where it usually contributes to transcriptional stability. However, its exact role in ncRNA should be further explored. With respect to tRNA, this is one of the most observed modifications and has an important role in its stability and function.

The 16S rRNA residues pseuduridylation in mitochondria is required for mitochondrial protein synthesis and for overall cell viability and survival. Designed to determine the association of new metabolites with LV remodeling, this study examined 1,052 Bogalusa Heart Study participants and performed untargeted metabolomics analysis of fasting serum samples [163]. In the combinatorial and ethnic stratification analysis, the multiple linear and multinomial logistic regression models were used to detect associations between metabolites and LVMI and RWT, and LV geometric phenotypes were classified. In the entire sample, PU and N-formylmethionine were significantly related to LVMI. The current study identified a new correlation between N-formylmethionine and PU, indicating that the mitochondrial-derived metabolites can be used as early biomarkers for HF. Furthermore, association between two circulating secondary bile acids (glycolithocolate sulfate and glycocholenate) and the risk of AF has previously been determined among 1,919 blacks in the ARIC cohort [166]. After adjustment for multiple comparisons, analysis of 245 metabolites in the combined cohort detected PU, uridine, and acisoga as AF related metabolites. Taken together, the previously established prospective association between secondary bile acids, glycocholic acid sulfate, and the incidence of AF was replicated, and the nucleoside and polyamine metabolism were identified as potential AF markers [167]. In fact, pseuduridylation should be further studied to understand its role in aging driven CVDs.

### Uridylation

This is the process of adding non-template uridine to RNA end for regulation of certain cellular processes [168]. PolyU polymerases or Terminal Uridylyl Transferase (TUTases) are the enzymes that catalyze this process of uridylating 3' end of RNA. The uridylic nucleotides are derived from uridine diphosphate glucose (UDPG) in glycogen synthesis. It has been reported that uridylation of polyadenylated mRNAs can induce the exonuclease activity and promote that degradation leading to downregulation of their expression [169].

Previous studies have shown that uridine is incorporated in mammalian heart cells, and this incorporation leads to an increased level of free uridine in Langendorff-perfused hearts and *in vivo* [170]. Isolated rat hearts underwent 30 min of low-flow ischemia, and uridine was added to the perfusion medium during reperfusion for 30 min [171]. Creatine phosphate, uridine acid, adenine nucleotide, and glycogen were measured at the end of the experiment. Low flow ischemia resulted in 53%, 23%, and 15% degradation of creatine phosphate, adenosine triphosphate, and total adenine nucleotide, respectively, and 56% and 53% degradation of uridine triphosphate and glycogen during ischemia, respectively. Meanwhile, in the oxygenated hearts, uridine supply resulted in decreased creatine phosphate concentration and increased uridine triphosphate levels, but had no influence on UDPG, adenosine triphosphate, or glycogen concentrations. If the nucleoside was provided during reperfusion, it induced a complete recovery of myocardial ATP, total adenine nucleotide content, uridine acid concentration, and glycogen resynthesis to super-normal values.

Adenylate-uridylate (AU) rich elements in 3'UTR of many transiently expressed genes modulated mRNA instability and translation. CUGBP and ETR 3-like factor (CELF) proteins are the family of RBPs that play a dominant role in the RNA metabolism [172]. Using the acute myocardial infarction (AMI) animal model, it was found that the expression level of CUG triple repeat RNA binding protein 1 CUGBP1))/CELF1 in the heart damaged by AMI was reduced [173]. Further research suggested that two highly conserved AU enriched elements in CUGBP1 3'UTR account for the reduced expression of CUGBP1 [174]. Thus, the uridylation of CUGBP1 was observed to play a major role in heart disease and could form a novel post-transcriptional MI gene regulatory mechanism.

### Adenosine-to-inosine editing

Adenosine-to-inosine (A-to-I) RNA editing is a usual post-transcriptional modification, which plays an important role in preventing endogenous double-stranded RNA from erroneously activating innate immunity, and is related to various regulatory processes and diseases, such as autoimmune, cardiovascular diseases and cancer [175]. In addition, endogenous A-to-I editing mechanism has recently been used in RNA engineering [176].

### A-to-I in mRNAs

A-to-I RNA editing of adenosine deaminases act on RNA (ADAR) modified the mRNA and redirected it to translate a new protein and editing of mRNA encoding actin crosslinking protein filaggrin A (FLNA) mediated Q-to-R conversion in interactive C-terminal region [177]. One study indicated that human and mouse cardiovascular tissues underwent a lot of editing, and FLNA RNA was the most highlighted substrate [178]. RNA-seq data from patients indicated a significant decline in FLNA editing related to CVDs. Using mice edited with only damaged FLNA, increased vasoconstriction and diastolic hypertension were observed with increased phosphorylation of myosin light chains, arterial remodeling and LV wall thickening, which ultimately led to cardiac remodeling and decreased contractile output. Taken together, these results suggested the causal relationship between RNA editing and CVDs development, hinting that a single epigenomic RNA modification could sustain cardiovascular health. Meanwhile, the A-to-I RNA editing is catalyzed by the ADAR family enzymes and is important in transcriptome regulation of RNA metabolism. This study demonstrated that the cathepsin S mRNA (CTSS) encoding cysteine proteases correlated with atherosclerosis are highly edited in human ECs [179]. The 3' UTR of CTSS transcript contains two reverse repeats, namely AluJo- and AluSx+ regions, which recognized by ADAR1 as an editing substrate. In ECs, the overexpression of ADAR1 or treatment with hypoxia or inflammatory induces CTSS RNA editing, thereby increasing the cathepsin S expression [180]. The level of ADAR1 and CTSS RNA editing are connected with variations in the cathepsin S levels in the atherosclerosis.

### A-to-I in ncRNAs

LncRNA has become a key regulatory factor in atherosclerosis, but the post-transcriptional regulatory mechanism involved in disease related lncRNA expression is not completely understood. These studies showed a more than two-fold increase in nuclear paraspeckle assembled transcription 1 (NEAT1) lncRNA expression in PBMC of CAD patients [181]. The NEAT1 expression was observed to be induced by TNF-α, and the NEAT1 silencing significantly reduced TNF-α -induced pro-inflammatory responses in the ECs defined by CXCL8, VCAM1, CCL2 and ICAM1 expressions [182]. ADAR1 overexpression up-regulated NEAT1 levels, whereas the ADAR1 silencing suppressed NEAT1 levels and TNF-α induced increases. Stable RNA binding protein AUF1 silencing decreased the level of NEAT1, while the ADAR1 silencing influenced the combining capacity of AUF1 to NEAT1 [183]. Overall, these results suggested a mechanism by which the ADAR1 catalyzed A-to-I RNA editing to control the stability of NEAT1 lncRNA in atherosclerotic cardiovascular disease.

A-to-I editing in the microRNA seed sequences can alter the miRNAs target set, thereby altering their function [184]. Using the common RNA sequencing data, A-to-I edited vasoactive miRNAs were identified. The A-to-i editing of pri-miRNAs in the vascular ECs and fibroblasts was quantized and increased continuously beneath ischemia. Mature miRNA edits with the highest miRNA expression were identified, that is miR-376a-3p, miR-376c-3p, miR-381-3p, and miR-411-5p. These results indicated that both ADAR1 and ADAR2 could edit pri-miRNA, and the miRNA editing was also enhanced in the mouse hind limb ischemia model and isolated human venous ischemia [185, 186]. In summary, the A-to-I editing of microRNA was a universal phenomenon observed in ischemia, and each editing generated a new microRNA with a particular targeting group, thereby increasing angiogenesis. Further research needs to be done to identify other targets of this modification.

### N4-acetylcytidine (ac4C) RNA modification

N4-acetylcytidine (ac^4^C) was detected as a highly conserved RNA modification of cytidine in the non-coding RNA such as tRNA and near the translation initiation codon of mRNA [187]. Currently, research has revealed that the presence of ac4C at m7G Cap level in the human mRNA and its underlying effect in ameliorating mRNA stability. The ac4C modification is usually carried out with the help of N-acetyltransferase 10 (NAT10), tRNA acetyltransferase (TAN1) and certain snoRNA. The ac4C is related to the occurrence and progression of multiple cardiovascular diseases. The NOD-like receptors activation by ac4C stabilized transcripts stimulates the inflammatory cytokines production and increases oxidative stress *in vivo* [188]. According to certain reports, there is a strong correlation between ac4C levels and inflammation induced by the IL-1β/pyrin domain comprising three NLRP3-mediated signaling pathways in atherosclerosis, MI, AF, and I/R injuries [189, 190]. Therefore, ac4C RNA modifications could be an important contributing factor in the pathophysiology of CVDs and should be further investigated.

### 2'-O-methylation

2'-O-methylation (2' O-me/Nm) is defined as methylation of ribose at the 2-OH group and is one of the most usual RNA modifications. Because it can modify any nucleotide base to produce 2'-O-methyladenosine, 2'-O-methylcytidine, 2'-O-methylguanosine and 2'-O-methyluridine, collectively known as Nm. The 2'O-Me/Nm had been widely researched in the tRNA, rRNA and snoRNA, and latterly been reported in mRNAs as well.

### 2' O-me in mRNAs

Nm is one of the most plentiful RNA modifications quantized after PU. Though found more commonly in mRNA cap, it has also been surveyed in internal coding sequences (CDS). The mRNA 2'O-Me is actively involved in the regulation of gene expression and may adjust these protein levels. Cap methylation is usually carried out by two enzymes - CMTR1 and CMTR2 that 2′-O-methylates at the first and second transcribed nucleotide of the mRNA respectively. There have been reports of increased expression of CMTR1 in mice models of the neurodegenerative Alzheimer’s disease (AD). This mRNA modification is also thought to play a role in splicing. Therefore, Nm can adjust a variety of biological and pathophysiological processes including CVDs [191, 192].

### 2' O-me in ncRNAs

rRNAs are the ncRNAs that undergo most of Nm modification while this modification has also been observed in snoRNA and tRNAs. It has been observed that with respect to adult heart tissue, developing tissues undergo lesser methylation in specific regions as specific patterns of methylation occurs during development [192]. These changes affect the ribosomal function, effect being observed on the translation process and consequently in CVDs development. According to recent research, the snoRNA-guided 2'O-Me regulated the mRNA transcripts, suggesting that the snoRNA-guided 2'O-Me affected cardiac development and resulted in HF [193]. There are also reports that suggest that in animals, piRNA is Nm modified at its 3' terminal to avoid polyurination and prevent its degradation by exonucleases. It is supposed that by methylating piRNAs, these modifications could have an impact in CVDs.

### Modifications of U34 on tRNA

This is a modification of the 34^th^ position wobble Uridine (U34) on tRNA and this modification occurs post-transcriptionally to ensure proper functionality of the tRNA. Such aberrant modifications have been observed in mt-tRNA leading to the development of hypertrophic cardiomyopathies and lactic acidosis. This is commonly known as mitochondrial dysfunctions usually associated with aging disorders, wherein mitochondria-nuclear signaling pathways are activated. In this study, the mutant mitochondrial (mt)-tRNAs^Leu(UUR)^ was observed to lack the taurine containing modification of wobble uridine (U34) normally present in the wild-type UUR [194]. This data suggests that mt-DNA diseases could directly influence microRNA expression [195]. In addition, it has been proven that modifications on the mt-tRNAs were dynamic, a cellular answer to stress by regulation of mt-tRNA modification enzymes expression. There have been several studies that have reported that U34 modifications of tRNA modifying enzymes such ELP2 and ELP3 lead to neurodegenerative disorders and even ALS as evidenced in several animal models [196]. U34 modification seems to be important to protein processing due to its position and any impedance to this modification can cause protein aggregation as observed in several aging associated disorders [197].

## CONCLUSIONS

Dynamic RNA modification is quite abundant in most RNA species such as mRNA, rRNA, and tRNA and have been proved to be critical in cellular functions. This review attempted to consolidate different aspects of RNA modifications that are crucial in regulating aging-associated CVDs. We first briefly summarized the different types of RNA modifications commonly abundant in cells as well the different ncRNA types. We then enumerated the different methods of identifying the RNA modifications. As sequencing techniques were one of the most superior methods of identifying RNA modifications, we also summarize the different bioinformatic tools and databases that help in their study. We then summarized the role of different modifications in various aging-driven CVDs. These observations have augmented our current understanding of the effect of epigenetics on cardiovascular aging and disease that has tremendously increased during the last few years. Therefore, epigenetic modifications like chromatin remodeling, DNA occupation, and changes in ncRNA expression, which are driven by modifications in RNA bases, contribute to the progression of cardiovascular aging and disease. However, one of the major challenges in the field is the difficulties in detecting these modifications by sequencing methods. Despite improvements in the field, there is still an urgent unmet need in developing advanced tools/techniques to detect and study these RNA modifications, especially with respect to precise profiling of these modifications. Unfortunately, a non-uniform detection of these modifications also poses a disadvantage of not accurately representing the profile of RNA modifications. It is certain that more work needs to be done to understand the reason behind such dynamic modifications and pathogenesis, ultimately moving further towards pattern recognition in aging-driven diseases. There is no doubt that unraveling such crosstalk between RNA modifications and other DNA or RNA modification methods, will further deepen our understanding of these mechanisms and potential therapeutic strategies for CVDs. In the future, further research enhancing the understanding of RNA epigenetic mechanisms, facilitating the development and use of epigenetic modification therapies to improve clinical outcomes for heart disease and other age-related diseases is needed.
